# Twist removal of healed vs. nonhealed implants—a mechanical and histological study in mini pigs

**DOI:** 10.1186/s40729-016-0059-x

**Published:** 2016-11-25

**Authors:** Ricardo de Oliveira Silva, Fabrício Passador, Paulo Henrique Ferreira Caria

**Affiliations:** 1 FOP/UNICAMP, Piracicaba, SP Brasil; 2 CPG São Leopoldo Mandic, Campinas, SP Brasil

**Keywords:** Removal torque, Peri-implant bone, Dental implant, Mini pigs, Reverse torque

## Abstract

**Purpose:**

The objective of this study was to evaluate the effect of removal torque (reverse torque) of titanium implants in peri-implant bone.

**Methods:**

The P1-M1 teeth were extracted bilaterally of 6 mini pigs (BR-1). Each animal received 6 titanium implants, three for each side of mandible. On the right side of mandible, 3 implants reminded 9 months (9M) under masticatory activity and on the left side, other 3 implants were placed and immediately removed (IR). All 36 implants were removed by removal torque, and the recorded values were statistically analyzed. Animals were euthanized right after the removal torque and recording. Each third (cervical, medium, and apical) of peri-implant bone was extracted and analyzed histological and immunohistochemically. Student’s *t* test was used to determine statistical differences in the values between the 9M and IR samples. Data were presented as means with standard deviations. The level of significance was set at 5% (*P* < 0.05).

**Results:**

Removal torque was higher in 9M experimental situation than in IR. Histological characteristics of mature bone were presented in the 9M experimental condition, and immature bone characteristics were presented in the IR experimental condition. Removal torque caused small fractures and rounding in the bone grooving. Immunohistochemical analysis reinforced the histological results; Student’s *t* test provided statistically significant differences to osteocalcin expression in 9M samples and no statistically significant differences expression to collagen I in both experimental conditions (*P* < 0.05).

**Conclusions:**

Removal torque caused microscopical fractures and smoothing in the peri-implant bone grooves, but it does not compromise the bone healing.

## Introduction

Since the discovery of osseointegration by Branemark in Sweden in 1960, where found that when titanium screws left undisturbed in bone, the osteocytes grow in close apposition to the titanium surfaces and provide firm anchorage. This discovery was successfully applied in dental and craniofacial reconstructive surgery in 1965 [1, 2]. Dental implants became a common procedure in the modern dental treatment with long-term success rates exceeding 90% reaching up to 100% [3, 4] due to the development of some implant systems [5]. However, the increased use of dental implants also improved the fails. The main causes of failure are incorrect position, fracture, peri-implantitis, chronic diseases, and smoking [6–9].

Several studies indicated that screw loosening appeared to be one of the most common complications in dental implants once osseointegration has occurred, especially in single-tooth implant restorations [8, 10, 11]. The incorrect position of implants can cause maxillary sinus membrane damage, pressure on the dental nerves, or difficulties in prosthetic procedure as well as inconvenient esthetical problems. Esthetical requirements of patients have increased, especially for anterior teeth [12, 13]. Even after successful osseointegration, the implant remotion may be necessary [1, 12, 14, 15].

To correct the wrong position or fractured implant is necessary to remove it. For this purpose, it may be used various surgical techniques such as the use of trephine, implant drills, ultrasound, and others. But the use of these techniques cause great loss of peri-implant bone, what limits or prevents a new immediate rehabilitation [16, 17]. Alternatives to removal implants without losing or expanding alveolar bone led Anitua and Orive [18] to use the counter torque. Studies comparing counter torque with trephine drills to remove implants indicated better performance for the first [18, 19].

The causes of implant failure are well known and described; however, what happened with the peri-implant bone that can influence on the success of a reimplantation needs to be better described, with the increase of implant removals to replacement for functional or aesthetic corrections and the need to reduce alveolar bone loss [20].

Many authors investigated bone reactions around dental implants [17–21]. What happens in the peri-implant bone implants removed is not reported in scientific articles. This study evaluated the peri-implant bone after his immediate removal and after 9 months of osseointegration. The aim of the present study was to evaluate the peri-implant bone after dental implant removal.

## Materials and methods

### Animals and preparation

This study was approved by the University Animal Ethics Committee-CEUA/UNICAMP-(Campinas, SP) (no.2730-1/12). Six adult male mini pigs (BR-1 mini pigs, São Paulo, Brazil) with ~36 months old and weighed ~55 kg were used in the experiment. The mini pigs were kept in the Experimental Center of the Veterinary Faculty (FESB-Bragança Paulista, SP) and were allowed to adapt to the environment 1 week prior to surgeries. At the beginning of the study, all animals underwent a physical examination by a veterinarian and were found to be healthy. During the study period, the mini pigs were weighed if abnormalities in food intake were observed [21]. The identification of the animals was enabled by marking earrings numbered. The mini pigs were kept separately in cemented stalls. Fresh water was available ad libitum. For 12 h, before surgery, the animals were fasting with water ad libitum. The animals were inspected after the first few postoperative days for signs of wound dehiscence or infection and weekly thereafter to assess general health.

The removal torque and the histological and immunohistochemical analysis of peri-implant bone were conducted in the mandible of the mini pigs.

The animals were premedicated to induce anesthesia with midazolam (0.2 mg/kg) (Medley, Sumaré, SP, Brasil) and chlorpromazina IM (0.1 mg/kg) (Cristália, Itapira, SP, Brasil). An endotracheal tube was used for intubation, and a mixture of isofluran (Baxter Healthcare Corporation, IL, USA) with oxygen in a ratio 1:1 (5–10 mL/kg/min) was used to maintain anesthesia during the experiment. Local anesthesia was performed with lidocaine 2% with epinephrine 12.5 μg/mL (Xylocain/Adrenalin®, Astra, Wedel, Germany). After surgery, veterinarian Pentabiotic Reinforced antibiotic 40.000 UI/kg (Eurofarma, Itapevi, SP) and anti-inflammatory dexamethasone 3 mg/pig (MSD, São Paulo, SP, Brasil) were administered IM application .

### Surgical procedures and implant removal

**Fig. 1:**
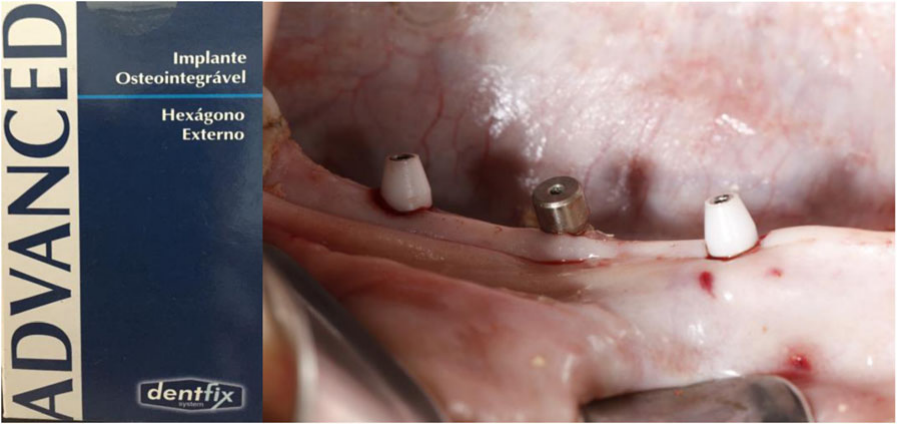
Type of implant and clinical picture of implants position in the mandible of mini pigs

**Fig. 2:**
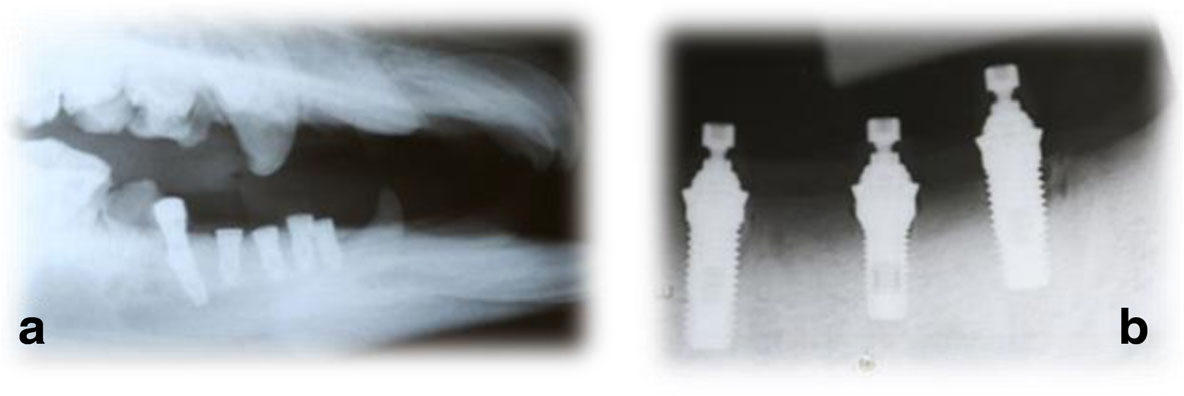
Radiographs illustrating implants in the mandible of mini pigs. **a** Radiography of the mini pigs head with implants in both mandible sides. **b** Periapical radiograph of implants position

**Fig. 3:**
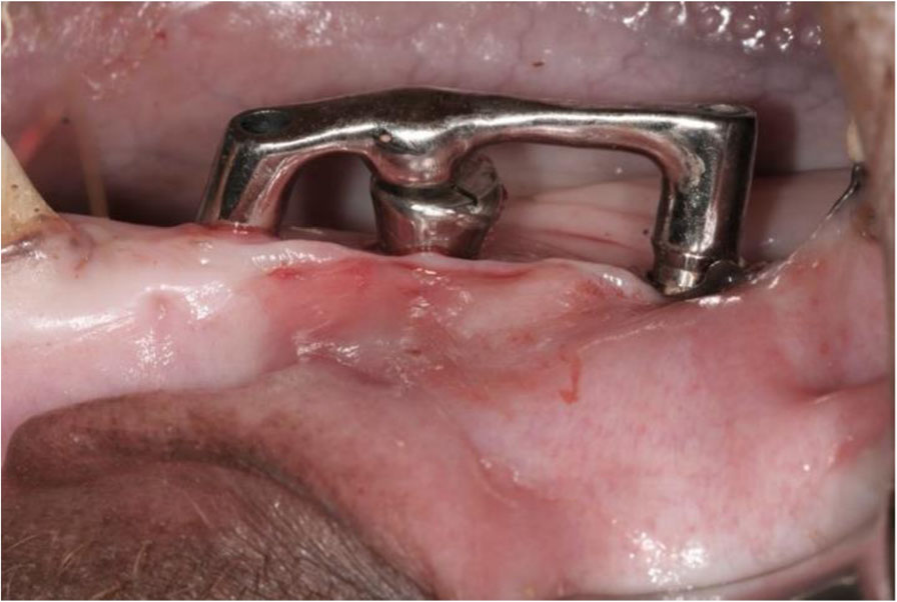
Prothesis fixed installed on the three implants

The same operator performed all the surgeries and radiographic. The P1, P2, P3, and M1 teeth were extracted bilaterally of each animal. The tooth extractions were difficult in every case because the roots were divergent and usually curved distally. It was necessary to odontosection before extracting them [22]. After 4 months of healing, three external hexagon implants (EH) (Dentifix. Santa Rita do Passa Quatro, SP, Brasil) with the same diameter and length (ø4.1 × 10 mm) were placed with the STA surface on one side of the mandible (Figs. 1 and 2). The side was chosen by lot. Six months later, this implant group received a prosthetic (Fig. 3) to improve the bone tension [23]. Three months later, the 3 implants with prosthetic were removed, totalizing 9 months (9M) and opposite side of the mandible three new implants were placed and immediately removed (IR). Each miniature pig received 6 implants, 3 on each side of the mandible. Thus, a total number of 36 implants were placed. Pigs were anesthetized as described above; all dental implants were carefully removed by a counterclockwise force (removal torque) with a torque driver (Retriever Maximus - Belo Horizonte, MG, Brasil); and the level of torque required to remove the implant from the bone was recorded by mark-10 universal torque series sensor STW, and removal torque were read by a force/torque indicator model BGI (JLW Instruments, Chicago, IL, USA). Afterwards, anesthetized pigs were euthanized with pentobarbital; their mandibles were cut and the respective peri-implant bone was removed in small blocks (10×10×6 mm).

### Histology and immunohistochemical analysis

The mandibles were sectioned into left and right segments, and each peri-implant bone was sectioned again to individualize them. Each peri-implant bone block was fixed in buffered formalin solution, pH 7.0, for 6 days, demineralized in 10% formic acid, and dehydrated through progressing alcohol concentrations and paraffin-embedded. Paraffin blocks were sectioned at 7-μm thick mounted on poly-l-lysine coated glass slides (Sigma–Aldrich, Gillingham, UK) and processed for hematoxylin-eosin staining and for immunohistochemical analysis. Each peri-implant bone paraffin block was longitudinal and colored with hematoxylin-eosin (H&E) to identify sites of new bone destruction or remodeling.

Immunohistochemical analysis was performed on duplicate tissue sections of peri-implant bone from each experimental specimen (9M and IR—randomly chosen). Sections were deparaffinized and rehydrated by rinsing with xylene for 10 min, industrial methylated spirit for 5 min, and more 5 min in tap water. In order put out endogenous peroxidase activity, sections were incubated at room temperature in 3% hydrogen peroxide for 10 min. Two proteins were evaluated due to their sequential expression during bone healing. Collagen type I because it is expressed early in the healing process [24]. Osteocalcin because it is a late marker of bone formation and is expressed during mineralization by osteoblastic cells [24]. To prevent nonspecific protein binding, serum-free blocking agent (DAKO, Hamburg, Germany) was used. The sample was allowed to react for 1 h at room temperature with a primary anti-collagen I antibody (Abcam, Cambridge, UK) and anti-osteocalcin antibody (Takara Biomedicals Europe, France). Immunohistochemical analysis was performed at different thirds of the peri-implant bone (cervical, medium, and apical). Each third was selected at least two times per sample and analyzed. Sample images were captured then observed by means of Leica DM 4000 light microscopy (Leica Cambridge Ltd, Cambridge, UK) incorporating a Leica DFC 320 camera (Leica Cambridge Ltd) for computerized images in histological and immunohistochemistry analysis with a ×40 magnification.

### Image and statistical analysis

Hematoxylin-eosin-stained section images were digitized and analyzed in order to recognize the presence of native bone tissue by the presence of osteocyte lacunae-containing cells and the newly formed bone tissue recognized by the absence of lacunae. Also, the characteristics of peri-implant bone, presence or absence of bone fractures, and the shape and contour of bone grooving resultant of the trephine action were analyzed. Histological analysis was performed in images of the semi-serial slices of each peri-implant bone. They were captured by a digital camera (Samsung, South Korea) coupled to a light microscope (Zeiss, Germany) with original ×200 magnification and resolution of 600 dpi. Images around 116–80 cm were captured of each third of the peri-implant bone. Then, a digital framework of entire peri-implant bone was built by the combining three images.

Immunohistochemical analysis also was performed on three thirds on each sample with collagen I and osteocalcin. The same image capture and construction were made, but they were measured, and the value was defined by the positive-staining samples and was used to automatically analyze images of all samples that were stained under identical conditions for both proteins and implant removals.

In the analysis of both mandible sides, the images were acquired at ×200 magnification using a Nikon E600 microscope (Nikon Instruments Inc, Melville, USA). The integral optical density (IOD) of target protein was measured with Image-Pro Plus 5.0 (Media Cybernetics, Rockville, MD, USA). In the process of measurement, the values were defined firstly by determining the positive staining of control sections and were used to automatically analyze images of all samples that were under identical conditions (u/pixel) [25].

Statistical analyses were performed with SPSS software (SPSS, Chicago, Ill). Student’s *t* test was used to determine statistical differences in the values between the 9M and IR samples. Data were presented as means with standard deviations. The level of significance was set at 5% (*P* < 0.05).

## Results

### Clinical observation

No remarkable complications were found during the healing period. At sacrifice, all 18 implants fixed after 9 months were considered successfully integrated at the time of the removal and none showed any mobility or signal of infection at sacrifice. There was no difference in the healing between animals who had the implants immediately removed after installation, and animal whose implants were removed 9 months later of installation.

### Removal torque

The mean and standard deviation of removal torque are illustrated in Tables 1 and 2 for both experimental specimens. The removal torque values increased after 9 months, with significant differences between IR and after 9M specimens.

**Table 1:** Removal torque value (Ncm) of three implants immediate removed (IR) per animal

Animal	Mean	Standard deviation	Minimum	Maximum
1	98.3	5.5	92.2	103.3
2	91.6	9.1	82.1	102.5
3	105.3	8.3	100.4	115.0
4	71.6	10.5	61.2	82.2
5	78.6	5.8	72.7	83.1
6	88.6	6.6	81	93.6

**Table 2:** Removal torque value (Ncm) of three implants removed after 9 months (9M) per animal

Animal	Mean	Standard deviation	Minimum	Maximum
1	150.1	30.2	122.7	184.4
2	163.3	35.1	132.4	205.3
3	175.2	15.2	153.2	204.6
4	163.6	15.4	157.3	185.1
5	153.3	15.2	146.2	174.2
6	150.3	26.4	129.2	174.6

### Histological analysis

**Fig. 4:**
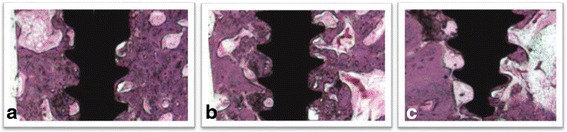
Representative photomicrographs of each third of the peri-implant bone of 9M experimental condition (H&E, ×40). **a** First third (cervical third). **b** Intermediate third. **c** Apical third. Bone grooving with no altered contour

**Fig. 5:**
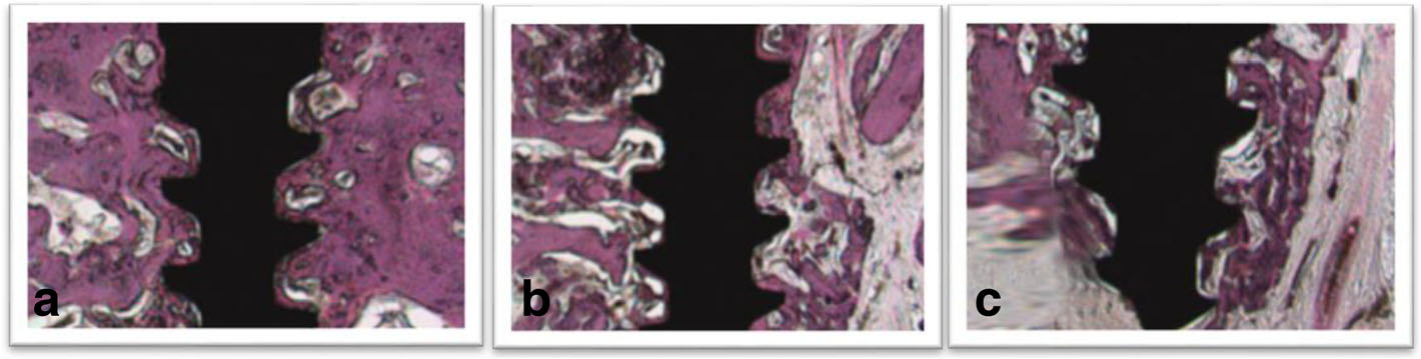
Representative photomicrographs of each third of the peri-implant bone of IR experimental condition (H&E, ×40). **a** First third (cervical third). **b** Intermediate third. **c** Apical third. Note the edges of bone grooving present rounded contour, mainly in the last third

Each third of the peri-implant bone was evaluated and showed not representative difference in the bone conditions for each experimental specimen separately (9M and IR) (Figs. 4 and 5). Removal torque did not alter the characteristics of mature bone and the healing process; thereby, did not cause significant damage in the peri-implant bone. After surgical trauma, it was possible to notice inflammatory process, which blood cells in the alveolar bone of IR specimens. At the 9M specimens, mature bone was evident, as well as presence of fibrous connective tissue without evidence of inflammatory infiltrate. A vital bone with many osteocytic lacunae was observed on the grooving of the internal wall of peri-implant bone. Many capillaries were present, and a rim of osteoblasts was observed on the bone margins. Natural inflammatory and bloody cells were visible only in IR specimens. As well as only in the IR specimens were observed small fractures and rounding in the bone grooving caused by implant trephine and removal torque. At 9M experimental condition, bone grooving presented clear contours, without rounding or fractures. In both experimental specimens, there was no evidence of bone formation particularly at tissue around the peri-implant bone surface. Only in the last third (apical) was possible to identify some bone fragments, probably caused by implantation procedure.

### Immunohistochemistry analysis

**Fig. 6:**
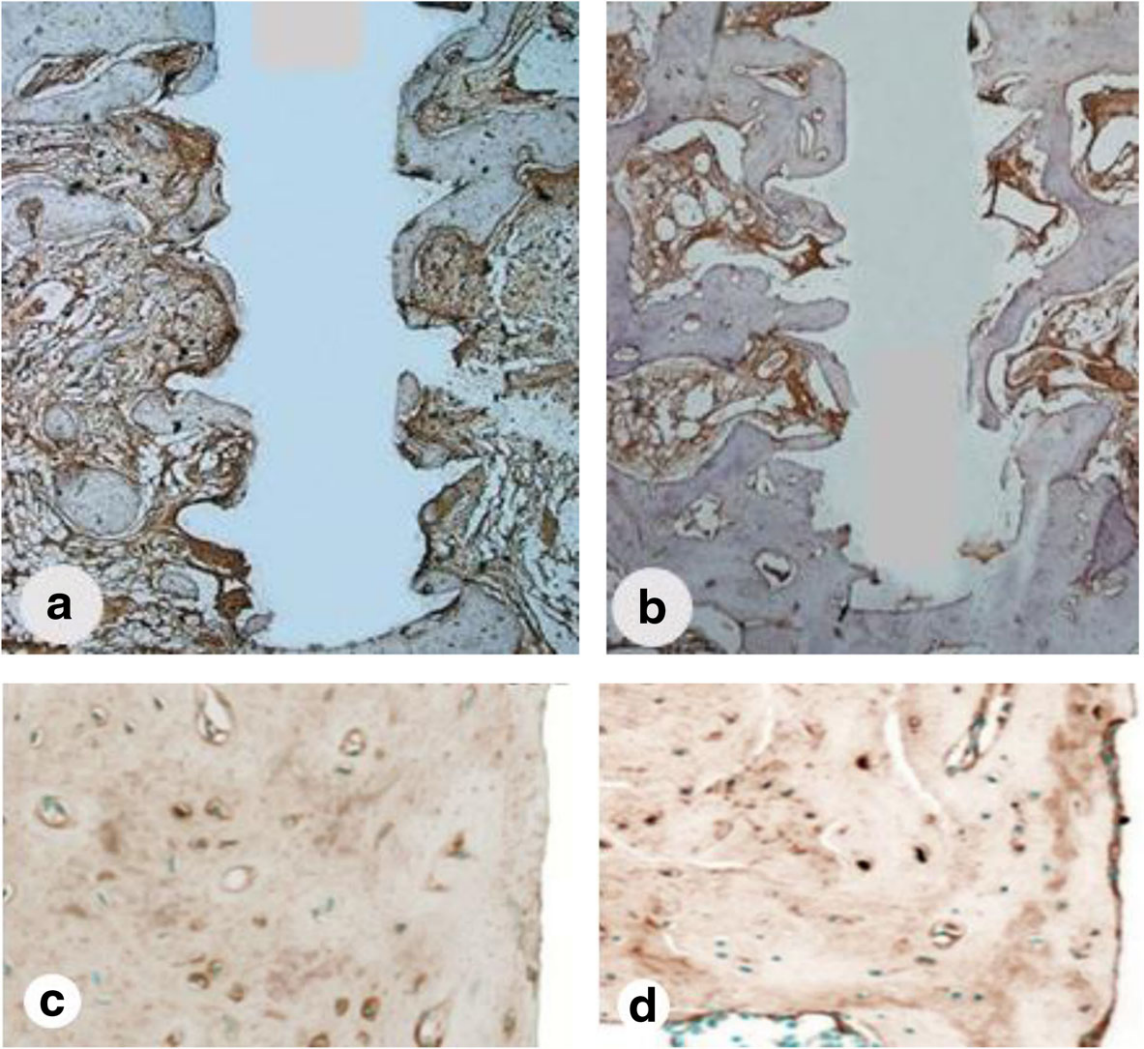
Immunohistochemical staining of osteocalcin (**a**) and collagen I (**c**) in sections from mini pigs mandible from 9M and IR osteocalcin (**b**) and collagen I (**d**). There were statistically significant differences to osteocalcin in 9M samples and no statistically significant differences to collagen I samples. Magnification: ×40 (**a**, **b**) and ×100 (**c**, **d**)

Duplicate sections of peri-impant bone were obtained from each implant sample to evaluate the percentage of stained areas in order to differentiate markers of collagen I and osteocalcin within both experimental conditions (Fig. 6). The highest collagen I expression values were observed at the IR experimental condition, and osteocalcin expression was higher at the 9M.

There was a statistically significant difference between the two experimental specimens (9M and IR) in immunohistochemical evaluation for osteocalcin expression (Tables 3 and 4). Immunohistochemical analyses allowed to identify manifestation of osteocalcin protein in all thirds of peri-implant bone in both models evaluated, with greatest expression to model which the healing time was higher (9M). Statistical difference presented was observed especially in the middle and lower thirds. The first third presented a difference, but it was not significant. The evaluation of collagen I expression did not show statistical differences. In all evaluated thirds, the presence of the protein was equivalent.

**Table 3:** Data showing the expression of osteocalcin in both experimental situations 9M and IR. Osteocalcin values considered (u/pixels) (*P* < 0.05)

	9M		IR	
Third	Mean	Standard deviation	Mean	Standard deviation
1o	190	3.6	110	10.2
2o	238*	6.1	120	10.6
3o	208*	7.2	90	11.1

*Statistically significant difference to osteocalcin expression to 9M experimental condition (*P* < 0.05)

**Table 4:** Data showing the expression of collagen I in both experimental situations 9M and IR. Collagen I values considered (u/pixel) (*P* < 0.05)

	9M		IR	
Third	Mean	Standard deviation	Mean	Standard deviation
1o	88.2	10.8	98.3	4.4
2o	90.5	10.2	100.2	7.2
3o	90.4	9.1	102.7	6.4

## Discussion

Dental implant revolutionized oral rehabilitation, becoming the natural teeth replacement by a titanium implant, a successful alternative to treat total or partial edentulism [14, 26, 27]. Nowadays, dental implants are definitely a current procedure in many dental offices [3, 28, 29]. Despite the long-term success shown by different studies [14, 30], implant failure is inevitable [31–33]. Since, to correct early or later failure implants is necessary to remove them, any tool available is necessary. Five different techniques to remove failing implants provided to be successful; however, the counter torque technique, used in our study, is the highest predictability for the insertion of another implant [17, 18, 34–36].

Previous in vivo assessments of bone healing around implants presented histological observations such as bone-implant contact studies under monitored torque values [19, 22, 37]. This study adds an extended methodology of previous investigations, because it provides beyond histological analysis and immunohistochemical analysis to assess peri-implant bone behavior in a real clinical condition.

Histological analysis of early failed implants has indicated that bone overheating might be the most probable cause of failure [33, 37–39]. Bur-forceps, neo bur-elevator-forceps, trephine drill, and scalpel-forceps are safe implant removal techniques, however, require experience and training of the operator. Counter torque technique is an easy and practice tool because it is a heating control procedure; it does not require training and can be performed by a beginner operator, so we opted to test this tool.

The clinical observations of this study showed all 18 implants fixed after 9 months were considered successfully integrated at the time of the removal, and none showed any mobility [40] or signal of infection [21, 33, 41, 42] at sacrifice.

The results of this work showed higher values of removal torque in 9M than in IR specimens. It was expected since the longer healing time (9M) promotes better osseointegration than immediate implant removal. It was verified by the presence of mature bone in the peri-implant bone in the 9M specimens [4, 22, 43].

In order to better use a model which reproduce the natural conditions of dental implant in action, minipigs (BR-1) have been used in this study [44], the nonprimate animal model that is most appropriate for the study of human mastication [45] and commonly used in research because suine and human share important anatomic and physiologic characteristics [46, 47].

The osseointegration process is quite similar to the primary bone healing [1]. After surgical procedure, there is an inflammatory process with local circulatory alteration. Afterwards, regeneration happens than bone tissue beginning to be replaced [1, 48]. As well as other peri-implant response happen, as the presence of collagen layer between bones and implant surfaces. The connective tissue consists in parallel collagen fibers supported by blood elements, setting the anatomical organization of collagenous ligament [49, 50]. All histological events described above were clearly observed in all IR specimens evaluated on this study. To the 9M specimens, those events were less evident due to postsurgical time.

At the 9M specimens, mature alveolar bone was evident. There is a presence of a fibrous connective tissue with no evidence of inflammatory infiltrate. A vital bone with many osteocytic lacunae was present around the grooving implant surface. Many capillaries were present, and a rim of osteoblasts was observed on the bone margins. Natural inflammatory and bloody cells were visible only in IR experimental condition.

As all surgical procedures of our study were taken with a strict care, there was no fracture or heating in bone tissue, which could compromise the results of this study. Long-term studies indicated in histological analysis of early failed implants that bone overheating might be the most probable cause of failure [33, 37–39].

The histological analysis also presented small fractures and rounding in the bone grooving caused by implants only in the IR condition. Considering the time healing in both specimens (9M and IR), after surgical procedure, some fractures and fragments were produced and those aspects were not presented after 9 months due to healing time. Removal torque caused little fracture and smooth on the peri-implant bone grooves just after the installation procedure (IR); however, none considerable damage or alteration compromised the bone healing. As at 9M specimens, the bone grooving presented clear contours, without rounding or fractures, demonstrating that removal torque is not a factor of dental implant failure. Even though some bone fragments were presented in the last third (apical) just in the IR procedure, it also did not compromise the bone healing.

According to Christenson R.H. [24], the bone structure, metabolism, and regulation are reflected by markers of resorption, formation, and/or turnover. Among the markers of bone resorption is the type 1 collagen degradation and maker of bone formation: Osteocalcin. Bone formation markers derive from the osteoblastic activity, formed during the different stages of osteoblasts proliferation, differentiation, and osteoid synthesis [6, 51–53], namely the bone osteocalcin, alkaline phosphatase, and other makers. Osteocalcin is expressed during mineralization by osteoblastic cells. [24, 54–56]. Those evidences supported us to analyze the expression of bone extreme activities: resorption (collagen I) and formation (osteocalcin). Our immunohistochemistry results expressed the bone repair because it showed higher expression of osteocalcin at the 9M specimens. Since the titanium implants were fixed for 9 months, peri-implant bone was submitted to masticatory tension [23] and that causes bone activity, stimulating osteocalcin expression, because it occurs during mineralization. Notwithstanding, collagen I expression did not show statistical difference between both experimental conditions, in spite of all numerical values were higher to IR experiment. It can also be explained by the healing time evaluated. Immediate implant removal caused histological evidence but has no time enough to express changes in the expression of collagen type I. The healing time was not extended because immediate removal represents a clinical situation in titanium implant procedures, when failure is detected just after its installation. The higher numerical values of collagen I expression to IR experiment condition indicate more protein activity than 9M. It also represents no removal torque influence in the healing process leading to the understanding that this does not hinder the immediate installation of a new implant in the same socket.

## Conclusion

Implant removal torque should be higher to remove implants with long-time installation than implants removed immediately after installation. Although, removal torque causes microscopical fractures and smooth on the peri-implant bone grooves, it does not compromised the bone healing.
